# An Examination of Tinnitus and Cochlear Functionality in both Hearing Impaired and Normally Hearing Individuals. The Relationship between Tinnitus and Cochlear Functions in Individuals with Hearing Impairment and those with Normal Hearing

**DOI:** 10.1007/s12070-025-05617-y

**Published:** 2025-06-12

**Authors:** Pavlos Pavlidis, Vasilis Spyridon Tseriotis, Sophia Karachrysafi, Anna-Rebekka Staufenberg, Haralampos Gouveris

**Affiliations:** 1 Department of Otorhinolarhingology / Head & Neck Surgery, University Medical Center Mainz, Mainz, Germany; 2 Laboratory for Clinical Pharmacology, School of Medicine, Faculty of Health Sciences, Aristotle University Thessaloniki, Thessaloniki, Greece; 3 Laboratory of Histology-Embryology, School of Medicine, Faculty of Health Sciences, Aristotle University of Thessaloniki, Thessaloniki, Greece

**Keywords:** Audiometry, Tinnitus, TEN Test, Otoacoustic Emissions, Hair Cells

## Abstract

Purpose: The evaluation of peripheral hearing organ function is vital for determining the pathophysiology of tinnitus and for establishing appropriate treatment strategies. This research seeks to investigate cochlear functions in tinnitus patients, irrespective of whether they have hearing loss. Methods: In this study, participants suffering from tinnitus were divided into two groups: the first study group (SG-I) consisted of 20 individuals who had associated hearing loss (HL). In contrast, the second group (SG-II) included 20 individuals without hearing loss. A control group (CG) was formed with 20 normal-hearing participants who did not have tinnitus. The tinnitus-related discomfort levels were evaluated using the Tinnitus Handicap Inventory (THI). Alongside pure-tone audiometry, Otoacoustic Emissions (OAE) were conducted to assess cochlear functions and to identify any dead regions (DR). Additionally, Threshold Equalizing Noise (TEN) was employed in the evaluation. Results: The analysis revealed a threshold shift in subjects from SG-I when evaluated using Transient Evoked Otoacoustic Emissions (TEN), with these levels showing statistically significant differences from both SG-I and the control group (CG). Furthermore, SG-I exhibited both threshold shifts and distortion responses (DR) as indicated by TEN. In contrast, Transient Otoacoustic Emissions (TEOAE) did not present any significant differences between SG-II and CG. The findings for Distortion Product Otoacoustic Emissions (DPOAE) in SG-II demonstrated significant decreases in emission amplitudes at 6 and 8 kHz. Conclusion: Conventional testing methods are suitable for evaluating tinnitus patients with hearing loss. Nevertheless, for patients with normal hearing experiencing tinnitus, the use of additional assessments such as Otoacoustic Emissions (OAE) and Tone-Evoked Noise (TEN) enables a more precise evaluation of the peripheral hearing mechanisms. The limited or absent otoacoustic emissions (OAEs) observed in the SG-I group indicate a potential reduction or loss of functionality in the outer hair cells (OHC) at the tested frequency. In contrast, an increase in the thresholds of the tone-equalizing noise (TEN) test points to a possible dysfunction in the inner hair cells (IHC).

## Introduction

Tinnitus is defined as an auditory illusion or a phantom sound sensation that occurs in the absence of external auditory stimuli, indicating the perception of sound without any objective sound source (ANSI, 1969). This condition serves as a symptom that can adversely affect quality of life, lead to somatization disorders, or contribute to depression. Regardless of its origin, tinnitus is processed in the auditory cortex [1]. The global incidence of tinnitus among adults has been reported to be between 10% and 15%.

Tinnitus may be connected to dysfunctions at any level of the auditory pathways, but it often initiates in the cochlea. Jasterboff suggests that the stimulus originates in the cochlea, which then leads to abnormal activity in the central pathway, ultimately maintaining the symptom [2]. A variety of studies have examined the link between tinnitus and the impairment of the efferent auditory system by evaluating the suppression of otoacoustic emissions (OAEs). Findings indicate that even slight variations in cochlear function can be observed through OAE testing before significant alterations are evident in the patient’s audiogram. As a result, the analysis of OAEs and their suppression is critically important and has become progressively vital for the differential diagnosis of disorders that could result in tinnitus.

Numerous efforts have been undertaken to elucidate the mechanisms underlying tinnitus, particularly in relation to cochlear function [5–7]. Spontaneous otoacoustic emissions (OAEs) have been frequently documented in tinnitus-related research. A variety of studies have indicated that transient evoked otoacoustic emissions (TEOAEs) and distortion product otoacoustic emissions (DPOAEs) can serve as effective methods for detecting early hearing loss, even when such loss is not evident in an audiogram. The findings regarding OAEs in the context of tinnitus have been inconsistent. While some studies have reported diminished TEOAE or DPOAE amplitudes in individuals with tinnitus, others have observed heightened amplitudes of TEOAEs or DPOAEs in the same population. This suggests that OAE results associated with tinnitus reflect altered outer hair cell (OHC) functionality. Another assessment utilized to investigate cochlear functions, particularly the role of inner hair cells in tinnitus, is the Threshold Equalizing Noise (TEN) test. Similar to OAE findings, the results from TEN tests have also been contradictory. For instance, Weisz et al. [3] identified cochlear dead regions in 8 out of 11 tinnitus subjects (72.7%), whereas Thabet et al. [4] reported dead regions in only 3 out of 20 subjects (15%). Furthermore, Buzo and Carvallo [5] noted significantly elevated thresholds in TEN tests among tinnitus patients with normal hearing, even in the absence of dead regions.

We hypothesized that the outcomes of the TEN and OAE tests in tinnitus patients with normal hearing would vary from those observed in the control group. Thus, the primary goal of this study was to examine cochlear functions in tinnitus patients, regardless of the presence of hearing loss (HL). In this research, OAE was applied to assess the function of outer hair cells, whereas the TEN test was implemented to evaluate the activity of inner hair cells in the cochlear area.

## Methods

The study was approved by the local Clinical Research Ethics Committee (approval Nr…….). All principles of the Helsinki Declaration for research in humans have been followed. Written informed consent was given by all participants, after they have been instructed about the study procedures.

### Subjects

Participants with a minimum of six months of tinnitus in at least one ear were selected for the study. They were classified into two study groups based on the presence of hearing loss (HL). The first group (SG-I, *n* = 20, 13 males, 7 females, age 43,2 ± 2.7 years) included those with tinnitus and sensorineural hearing loss, while the second group (SG-II, *n* = 20, 11 males, 9 females, age 41,8 ± 3,2 years) comprised individuals with tinnitus who had normal hearing thresholds bilaterally. A control group was also established, consisting of normal-hearing individuals without tinnitus (CG, *n* = 20, 10 males, 10 females, age 42,6 ± 1,8 years). Individuals with retrocochlear pathology, neurological disorders, psychological issues, temporomandibular joint disorders, pulsatile tinnitus, history of ear surgery or conductive hearing loss were excluded from the study. Individuals having tympanogram other than A type (like As type, Ad type, B type, C type) were also excluded. The Tinnitus Handicap Inventory was used to assess the levels of discomfort associated with tinnitus and to outline the profiles of the subjects. There was no significant age difference between male and female participants (*p* > 0.05).

### Audiological Evaluation

An extensive anamnesis was conducted for all individuals involved in the study. Air-conduction thresholds were recorded in the frequency range of 125 to 8,000 Hz via TDH-39 headphones, while bone-conduction thresholds were measured from 500 to 4,000 Hz using a B71 bone vibrator paired with the Grason Stadler (GSI) Audiostar.

### Evaluation of Tinnitus

The subject’s background was assessed to identify the localization of tinnitus. To ascertain the frequency and pitch of the tinnitus, the technique proposed by Vernon was utilized. In this procedure, the patient is presented with two sounds of different frequencies (f1 and f2, where f1 is less than f2) and is asked to determine which of the two sounds is closer to their tinnitus experience.

In the context of loudness matching, patients are presented with sounds that fall within the frequency range of their tinnitus. By soliciting feedback on whether the sound is perceived as louder or softer, the range is refined to identify the closest matching loudness level. The evaluation process generally begins with the ear that is more severely affected in cases of bilateral tinnitus; however, if the tinnitus is perceived equally in both ears, the left ear is assessed first. For cases of unilateral tinnitus, only the affected ear is evaluated. If the patient describes the tinnitus as being perceived in the center of the head, these instances are classified as bilateral tinnitus. To assess the discomfort level of the subjects, the Greek version of the Tinnitus Handicap Inventory (THI) was utilized. The THI consists of 25 items, each with three possible responses, and the total score is used to determine the level of discomfort associated with tinnitus.

### Threshold Equalizing Noise Test

The application of TEN was conducted for all subjects at frequencies of 500, 1000, 2000, and 4000 Hz, employing a Grason Stadler (GSI) Audio Star Pro audiometer along with TDH39 headphones. In instances of bilateral tinnitus, TEN was performed in both ears, whereas for unilateral tinnitus, only the ear affected was tested. The determination of TEN levels was based on the hearing thresholds of the subjects, as described by Brian Moore et al. (2000) [7]. For hearing thresholds better than 25 dB, the TEN was set at 50 dB to avoid false positive responses. When thresholds were between 25 and 60 dB, the TEN was adjusted to 70 dB, and for thresholds worse than 60 dB, it was set 10 dB above the threshold. The maximum adjustment for TEN should not exceed 90 dB due to cochlear sensitivity [13]. Additionally, it has been proposed by researchers that TEN could be performed at various severity levels [8, 9].

### Otoacoustic Emission Tests

In this study, TEOAE and DPOAE assessments were performed on all subjects using the Autodynamics EZ Screen, version 26.41.0. TEOAEs were evaluated in ears with tinnitus in quick screen mode, applying a 260 sweep at an intensity of 84 dB SPL for 80 milliseconds. The TEOAEs were recorded at frequencies of 1000 Hz, 1414 Hz, 2000 Hz, 2828 Hz, and 4000 Hz, capturing both the signal-to-noise ratios (SNR) and the amplitudes of the responses. For DPOAEs, the amplitudes and SNRs were recorded at frequencies of 1001, 2002, 3003, 4004, 6006, and 7996 Hz, using a 2f1-f2 ratio and L1/L2 intensity levels of 65/55 dB SPL, with an average of 500 sweeps conducted.

### Statistical Analysis

The statistical analyses were conducted using the SPSS software package, version 23.00, on a Windows operating system. The results were assessed through visual representations, including histograms and scatter charts, as well as statistical methods such as the Kolmogorov-Smirnov and Shapiro-Wilk tests. Descriptive statistics were performed, with median and interquartile ranges applied to non-normally distributed variables, while the mean (X) and standard deviation (SD) were used for normally distributed data. The analysis also included the median and interquartile range, in conjunction with the Kruskal-Wallis test. For double post-hoc comparisons, the Bonferroni test was employed. When comparing two groups, an independent sample t-test was conducted if the data demonstrated a normal distribution. A significance threshold of *p* < 0.05 was considered indicative of statistical significance. For patients reporting tinnitus on both ears, each ear was examined separately.

## Results

### Demographic Data

The study groups comprised a total of 31 participants, with 16 individuals (7 females, 9 males) experiencing both tinnitus and hearing loss in Study Group I (SG-I), and 15 individuals (6 females, 9 males) suffering from tinnitus alone in Study Group II (SG-II). The Control Group (CG) included 21 subjects (12 females, 9 males) who exhibited neither tinnitus nor hearing loss. The average age of participants in SG-I was 37.2 years, while SG-II had a mean age of 28.73 years, and the CG had an average age of 24.42 years. The Tinnitus Handicap Inventory (THI) scores indicated a range of discomfort levels from 1 to 5 for both SG-I and SG-II, with no significant difference observed between the two groups (p: 0.14) (*p* > 0.05). In SG-II, the frequencies of tinnitus varied, predominantly affecting higher frequencies (4–12 kHz). Conversely, in SG-I, tinnitus was primarily identified at frequencies associated with hearing loss. Hearing loss configurations were characterized as sloping in 11 participants, flat in 4, and a 6 kHz notch in 1 participant. Based on Pure Tone Averages (PTA), moderately severe hearing loss was identified in 5 individuals, moderate hearing loss in 4, and mild hearing loss in 3. The remaining participants exhibited PTAs within normal limits, although sensorineural hearing loss was noted at higher frequencies. Upon reviewing the patients’ medical histories, it was determined that there were no chronic diseases present, and none of the hearing losses were congenital. The profiles and the demographic data are depicted in Table 1.

**Table 1:** Profiles and demographic data of participating data

	Number of Subjects	Mean Age	Localization of Tinnitus			Hearing Loss	
			Bilateral	Unilateral	Inside the Head	Degree	Type
Study Group I	20 (13 males, 7 females)	43,2 ± 2.7 years	4	14	2	Mild (10 out of 20	Flat (3/20)
						Moderate (6 out of 20)	Sloping (14/20)
						Severe (4 out of 20)	Notched (3/20)
Study Group II	20(11 males, 9 females age	41,8 ± 3,2 years	4	12	4	Normal	
Control Group	20 (10 males, 10 females)	42,6 ± 1,8 years	-	-	-	Normal	

**Table 2:** The level of change observed in group thresholds in the context of TEN noise exposure

Groups	*N*	Frequency (kHz)	Min. (dB)	Max (dB)	Mean (dB)	*P* values	
						CG	SG-II
SG-I	*N* = 20	0.5	0	20	5.82	0.002	1.00
		1	4	14	4.15.	0.001<	0.06
		2	4	14	4.22	0.001<	0.01
		4	0	20	6.3	0.001<	0.01
						CG	SG-I
SG-II	*N* = 20	0.5	0	8	3.94	0.02	1.00
		1	2	12	4.01	0.2	0.02
		2	2	10	2.01	0.9	0.002
		4	-4	12	1.93	0.2	0.002
						SG-I	SG-II
CG	*N* = 20	0.5	-10	8	2.15	0.002	0.022
		1	-6	8	3.81	0.001	0.24
		2	-6	6	0.24	0.001	0.9
		4	-2	4	-0.14	0.001	0.2

### TEN Test Results

The current research established that the criteria for identifying a differential response (DR) required the masked threshold to be at least 10 dB lower than the unmasked threshold. The study examined threshold alterations in the presence of tonal masking noise (TEN) across all frequencies. In both SG-II and the control group (CG), no subjects demonstrated DRs. In contrast, as expected, SG-I revealed two subjects with DRs. The first subject (#32), a 39-year-old, exhibited sloping bilateral sensorineural hearing loss and tinnitus, with all hearing thresholds elevated under TEN, and a DR detected at 4 kHz in the right ear. The second subject (#56), aged 25, presented with bilateral flat, moderately severe sensorineural hearing loss and tinnitus, showing DRs bilaterally at 0.5 kHz, 1 kHz, and 4 kHz.

### OAE Tests Results

In the evaluation of TEOAE and DPAOE tests, both the signal-to-noise ratio (SNR) and emission response amplitudes were assessed. Due to the presence of hearing loss among subjects in the SG-I group, either minimal OAE amplitudes were recorded or no OAE responses were detected at all. Only five participants from this group exhibited responses in the OAE tests, with amplitudes ranging from − 12.5 to 8.6 dB. Consequently, comparisons were made solely between SG-II and the control group (CG). The analysis revealed no significant differences in SNR and amplitudes at frequencies of 1000 Hz, 1414 Hz, 2228 Hz, and 4000 Hz between SG-II and CG. Furthermore, when examining the SNRs in the DPOAE test, similar to the findings in TEOAE, no significant differences were noted between SG-II and CG at frequencies of 1, 1.5, 2, 3 and 4 kHz. However, emission response amplitudes at 6 kHz and 8 kHz were significantly reduced in SG-II, with p-values of 0.030 and 0.015, respectively, indicating statistical significance (*p* < 0.05).

## Discussion

The evaluation uncovered a significant threshold shift in subjects from SG-I, as determined by Transient Evoked Otoacoustic Emissions (TEN). The results showed statistically significant differences from both SG-I and the control group (CG). Moreover, SG-I presented both threshold shifts and distortion responses (DR) as reflected in the TEN findings.

The cochlea is considered a primary source of subjective tinnitus. It serves as the organ responsible for both receiving and generating sound, and the same conditions that can lead to hearing loss may also instigate tinnitus. Factors such as exposure to loud noises, head injuries, and ototoxic substances are recognized as significant contributors to the onset of tinnitus. Recent hypotheses propose that a lack of synchronization between the outer and inner hair cells may play a crucial role in its emergence. The discordant dysfunction theory posits that outer hair cells are more susceptible to damage than their inner counterparts, indicating that an auditory lesion predominantly impacts the outer cells, thereby creating a functional disparity. This theory is bolstered by various studies that reveal diminished otoacoustic emission amplitudes (TEOAEs and DPOAEs) in tinnitus sufferers compared to individuals with normal hearing. Additionally, contemporary theories link spontaneous otoacoustic emissions (SOAEs) to tinnitus generation, as it has been observed that individuals with elevated SOAE levels are more prone to experiencing tinnitus, implying that spontaneous activity may heighten the risk of developing this condition [10, 11].

There is a general consensus that extensive damage to the auditory pathways can lead to hearing loss that is associated with tinnitus. It is acknowledged that the adverse effects contributing to hearing loss and tinnitus mainly target outer hair cells (OHC). Nonetheless, standard audiometric evaluations do not enable the determination of whether the damage affects OHC or inner hair cells (IHC) [12–15]. As a result, this research focused on patients with hearing loss and tinnitus who presented normal audiogram thresholds, employing tests to assess the functionality of both OHC and IHC. This methodology was designed to explore the validity of the current theory.

This study hypothesized that tinnitus patients with hearing loss (SG-I) would present with higher hearing thresholds in the TEN test relative to tinnitus patients with normal hearing (SG-II). The results obtained from the TEN test demonstrated significant differences between SG-I and both SG-II and the control group (CG). It was found that all participants in SG-I with tinnitus exhibited increased hearing thresholds. Moreover, within SG-I, two participants showed distortion product otoacoustic emissions (DPOAEs). Notably, otoacoustic emissions were either absent or only minimally detected in SG-I. The sensorineural tinnitus model links tinnitus to damage affecting hair cells, degeneration of spiral ganglia, and decreased outputs at frequency-specific nerve endings of the auditory nerve. This sensorineural hearing loss (SNHL) may involve damage to both outer hair cells (OHC) and inner hair cells (IHC). For instance, if a total hearing loss of 60 dB is recorded at a specific frequency, it is plausible that 40 dB of this loss is due to OHC damage, while 20 dB may be attributed to IHC damage.

The limited or absent otoacoustic emissions (OAEs) observed in the SG-I group indicate a potential reduction or loss of functionality in the outer hair cells (OHC) at the tested frequency. In contrast, an increase in the thresholds of the tone-equalizing noise (TEN) test points to a possible dysfunction in the inner hair cells (IHC). According to existing research, hearing loss due to OHC impairment generally does not surpass approximately 50 dB at lower frequencies and 65 dB at higher frequencies. However, although the hearing loss levels in this group remain below these thresholds, the noted rise in TEN test thresholds suggests that IHC damage may have occurred, even in the absence of a detectable distortion product (DP). Additionally, during the history-taking for SG-I subjects, it was observed that they first reported hearing loss, which was later followed by the emergence of tinnitus. It is well-documented that auditory deprivation can lead to hyperactivity in nerve fibers and auditory nuclei, thereby enhancing plasticity at the central auditory level. Input deprivation within the nervous system is acknowledged as a critical factor that can instigate neural plasticity. In cases of auditory plasticity, such changes can alter sound processing and result in hyperactivity, potentially contributing to the onset of tinnitus and hyperacusis within the central nervous system. Specifically, cochlear degeneration, which disrupts the neural output from the cochlea, may trigger or intensify the perception of phantom sounds associated with tinnitus.

The TEN test results for tinnitus patients with normal hearing revealed significantly lower thresholds across all frequencies, with the exception of 500 Hz, when compared to tinnitus patients with hearing loss (HL). In contrast, when compared to the control group, a higher threshold was noted at all frequencies, with a significant difference observed only at 500 Hz. The literature presents conflicting findings on this matter [8–10, 14, 16–19]. According to Moore et al. (2000), a threshold increase of 10 dB or more during TEN tests in the presence of noise indicates a “dead region” at that frequency [7, 15–17]. Conversely, if the threshold increase is less than 10 dB, it may suggest degeneration in that frequency region; even in the absence of a specific dead region, disruptions in the central auditory pathways or auditory neuropathy may be present [7, 20]. In the present study, while no dead region was identified in normal-hearing subjects with tinnitus, the observed increase in hearing thresholds during TEN could indicate cochlear degeneration and may serve as an early indicator of hidden hearing loss. The elevation of thresholds in the presence of TEN has been attributed to poor processing efficiency, potentially resulting from reduced neural synchrony or synaptopathy [16, 21]. It has been noted that stronger disruptive factors are required to damage inner hair cells (IHCs) compared to outer hair cells (OHCs). Reports indicate that even with up to 80% damage to IHCs, audiometric thresholds may remain unaffected in rare instances [22]. Some studies in the context of hidden hearing loss have suggested that a dead region could be detected in normal-hearing individuals with tinnitus [8]. The variability in study outcomes may be linked to the diverse underlying etiologies of tinnitus.

### OAEs

OAE testing has been used as part of the protocol of audiologic evaluations and is currently an important tool in studies on tinnitus. Several studies have demonstrated a relationship between tinnitus and abnormal OAEs. In a study involving normal-hearing patients, TEOAEs and DPOAEs were abnormal in 70.2 and 68.4% of the group with tinnitus, respectively, but in only 16.10 and 50% of those without tinnitus [23]. It has been proposed that tinnitus may be associated with a diminished amplitude of otoacoustic emissions (OAEs). Fernandes and Santos discovered that normal-hearing individuals suffering from tinnitus displayed lower amplitude levels across all frequency bands in both ears, in contrast to those without tinnitus [24]. Furthermore, Azevedo et al. identified a notable difference in unilateral tinnitus cases, revealing that the amplitude was significantly reduced in the ear experiencing tinnitus [25].The notable increase in abnormal otoacoustic emissions (OAEs) among patients experiencing tinnitus suggests a connection to cochlear dysfunction in the development of this condition, especially at higher frequencies of 6 and 8 kHz. The same authors indicate that the auditory perception associated with tinnitus may stem from a dysfunction in the efferent auditory pathway, resulting in a loss of modulation of outer hair cells (OHCs). This loss could lead to abnormal auditory pathway activity, which might be erroneously perceived as sound.

Besides the application of transient evoked otoacoustic emissions (TEOAEs), distortion product otoacoustic emissions (DPOAEs) were utilized to assess cochlear functionality, with a focus on outer hair cells (OHCs). The OAEs recorded in normal-hearing participants with tinnitus (SG-II) displayed minor differences from those in the control group. TEOAE amplitudes and signal-to-noise ratios (SNRs) were found to be similar in both cohorts. In contrast, the DPOAE results indicated significant reductions in emission amplitudes at 6 and 8 kHz among normal-hearing individuals with tinnitus. Recent studies corroborated these findings, revealing significantly diminished DPOAE amplitudes in this demographic. It is recognized that the harmful effects of noise, ototoxic agents, and metabolic imbalances can lead to damage in hair cells, particularly in the basal region of the cochlea [26–30]. This degeneration may result in a decrease in OAE amplitudes, which could potentially initiate tinnitus. Nevertheless, the TEN test was confined to a frequency of 4000 Hz, thus limiting the exploration of this assertion. Our results may provide valuable insights for subsequent studies. Our results agree with those of previous studies.

Besides the application of transient evoked otoacoustic emissions (TEOAEs), distortion product otoacoustic emissions (DPOAEs) were utilized to assess cochlear functionality, with an emphasis on outer hair cells (OHC). The OAEs recorded from normal-hearing individuals with tinnitus (SG-II) displayed only minor differences when compared to the control group. The amplitudes and signal-to-noise ratios (SNRs) of TEOAEs were consistent across both cohorts. Conversely, the DPOAE results revealed significant reductions in emission amplitudes at 6 and 8 kHz among normal-hearing participants with tinnitus [31, 32]. It is well-known that the adverse effects of noise, ototoxic agents, and metabolic imbalances can lead to hair cell damage, particularly in the cochlea’s basal region [32]. Such degeneration may result in lower OAE amplitudes, potentially initiating tinnitus. However, the TEN test was confined to 4000 Hz, which limited the exploration of this hypothesis. Our results may provide important insights into future studies.

### Limitations

A significant constraint of the study is the omission of higher frequencies. Another point that should be stressed is the number of participants in each group. This occurred due to the need to form homogeneous groups as much as possible. The number of patients reporting bilateral tinnitus in both groups was limited.

## Conclusion

The presence of tinnitus in combination with hearing loss reveals different results from the TEN and OAE evaluations, which substantiates the theory of hair cell degeneration in the cochlea, affecting both inner and outer hair cells. Conventional audiometric assessments are not adequate for evaluating tinnitus patients who have normal hearing; hence, it is vital to test high-frequency thresholds and utilize TEN and analogous assessments to determine the functionality of inner hair cells.

## Data Availability

The data presented in this study are available on request from the corresponding author.
